# A260 ACUTE PANCREATITIS IN JORDANIAN CHILDREN

**DOI:** 10.1093/jcag/gwab049.259

**Published:** 2022-02-21

**Authors:** E M Altamimi, B Droubi

**Affiliations:** Pediatric, Jordan University of Science and Technology, Irbid, Jordan

## Abstract

**Background:**

Acute pancreatitis is a uncommon disease in children. The difference between pediatric and adult pancreatitis limit the ability of extrapolating the adults data and magnify the need for further pediatric research.

**Aims:**

We aimed to describe the demographics, clinical presentation and findings of acute pancreatitis in Jordanian children. In addition, we discuss the diagnostic criteria of AP and compare the role of various labratory and radiological investigations in the diagnostic process in our cohort.

**Methods:**

This is a retrospective study involving children below 18 years of age, diagnosed with acute pancreatitis at King Abdullah University Hospital, a tertiary healthcare center in Irbid, Jordan.

Patient electronic records from 2011 to 2020 were manually reviewed and data collected into an excel sheet. The data included their demographics (Age, sex, age at first recurrence in case of recurrent pancreatitis) and clinical data (presentation, physical findings, laboratory and radiological investigations, interventions and outcomes). Those with insufficient data were excluded.

**Results:**

A total of 22 patients with 59 episodes of AP were identified. Of those patients, 11 (50%) were males, and 11 (50%) had only a single AP episode. The mean age during the first attack of AP was 10 years (SD = 3.6 y, N = 21) and the youngest was 3.45 years.

Of the 22 patients who had the first AP episode, 11 (50%) had at least one more episode of AP later on. In those who did have recurrence; The median duration between the first and second episodes was 2.7 months, IQR = 11 mo. The median length of hospital stay was 3 days, IQR = 4 days.

Abdominal pain was the most common presenting symptom of AP (96%). Nausea and vomiting were less common (67% and 50%, respectively).

The cause of pancreatitis was identified in 18 (82%) children, while 4 (18%) were labelled as idiopathic. The most common known cause in our cohort was congenital biliary-pancreatic abnormalities; namely choledochal cyst and pancreatic divisum (6, 27%) followed by genetic cause (4, 18%) and biliary pancreatitis (4,18%).

Abdominal ultrasound (US) was the most used diagnostic imaging (33 exams). The most common finding was bulky and enlarged pancreas in 9 exams. MRCP evaluation was available for 10 episodes; 3 exams showed a normal pancreas, while 2 confirmed the suspicion of pancreatic divisum, 1 suggested cholangitis. 1 visualized a small choledochal cyst that was undetected with neither US nor CT.

Development of peripancreatic fluid collection (pseudocyst) was seen in 4 patients, two of which underwent intervention, one was treated with endoscopic gastro-cystic drainage and the other was treated with surgical drainage. Of the 22 children and 59 AP episodes, only 1 died.

**Conclusions:**

Acute pancreatitis is uncommon in Jordanian children. Although the small number in our cohort, we reported different etiological spectrum compared to reported literture.

**Funding Agencies:**

None

